# Survival after the diagnosis of breast or colorectal cancer in the GAZA Strip from 2005 to 2014

**DOI:** 10.1186/s12885-018-4552-x

**Published:** 2018-06-04

**Authors:** Chiara Panato, Khaled Abusamaan, Ettore Bidoli, Mokhtar Hamdi-Cherif, Daniela Pierannunzio, Stefano Ferretti, Mahmoud Daher, Fouad Elissawi, Diego Serraino

**Affiliations:** 1 Cancer Epidemiology Unit, IRCCS Centro di Riferimento Oncologico, Aviano, Italy; 2 Ministry of Health, PHC, Training and Education Department, Gaza, Palestine; 3 Faculty of Medicine and Cancer Registry, University of Setif, Setif, Algeria; 4 Centro Nazionale di Epidemiologia, Sorveglianza e Promozione della Salute, Istituto Superiore di Sanità, Rome, Italy; 5 Dipartment Morfologia, Chirurgia e Medicina Sperimentale, Università di Ferrara - Registro Tumori Area Vasta Emilia Centrale, Azienda USL Ferrara, - Servizio Prevenzione collettiva e Sanità pubblica, Ferrara, Regione Emilia-Romagna Italy; 6 WHO Office. Occupied Palestinian Territory, UNDP Building, Elnasr Street, Gaza, Palestine; 7 Ministry of Health, Primary Health Care directorate, Gaza, Palestine; 8 Friuli Venezia Giulia Cancer Registry, IRCCS Centro di Riferimento Oncologico, Aviano, Italy

**Keywords:** Gaza Strip, Cancer survival, Breast cancer, Colorectal cancer

## Abstract

**Background:**

Within a dramatic socio-political context, cancer represents a growing health burden in the Gaza Strip. We investigated the survival experience of people diagnosed with breast (BC) or colorectal (CRC) cancer from 2005 to 2014.

**Methods:**

Data included 1360 BC cases (median age 55.1 years) and 722 CRC cases (median age: 59.5 years; 52.5% men) recorded by the Gaza Cancer Registry according to a standard protocol. Clinical information was available for cases diagnosed in 2005–2006 only. Survival probabilities were estimated by Kaplan-Meyer method, while hazard ratios (HRs) and 95% confidence intervals (CI), adjusted for age and sex, were computed to assess factors associated with the risk of death.

**Results:**

Five-year survival was 65.1% for women with BC and 50.2% for patients with CRC. Advanced age (> 65 years), stage, and grade increased the death risk. Full access to therapies was associated with a reduced risk of death as compared with patients who had limited access (HR = 0.26, 95% CI:0.13–0.51 for BC; and HR = 0.11, 95% CI:0.04–0.31 for CRC).

**Conclusion(s):**

The 5-year survival after BC or CRC in the Gaza Strip was in line with estimates from surrounding Arab countries, but it was much lower than in developed Mediterranean countries (e.g., in Italy or in Jewish people in Israel).

## Background

The Gaza Strip, a narrow land located in the southern part of the Occupied Palestinian Territory (OPT), is an overcrowded area with a population of 1.8 million people (i.e., 5000 persons per km^2^) [1]. Although most of the population in the Gaza Strip has a challengeable life, with a high rate of poverty - 74% of families were estimated to live below the poverty line [2, 3] - life expectancy at birth reaches 71.5 years in males and 74.4 years in females [4]. Cancer is the second most common cause of death, after cardiovascular diseases, and it accounts for 20% of the whole expenditure for drugs [5, 6].

In the OPT, two population-based cancer registries were established in 1996 by the Palestinian Ministry of Health (MoH) -one in the West Bank, and one in the Gaza Strip [4]. Given the geopolitical context of the Gaza Strip [7, 8], the data collection process cannot fully reflect the whole cancer burden in the area. As a consequence, the World Health Organization (WHO) has recently given support to the Palestinian MoH in improving cancer registration [8]. Of all cases recorded between 2005 and 2014, breast cancer (BC) was the most common cancer among women (26.0% -skin cancers included), while colorectal cancer (CRC) was the second most common cancer in men (9.7% of all cases).

The Italian MoH promoted the “EUROMED Cancer Network” with the general aim to support extra-European Union Mediterranean countries in the development of effective anti-cancer programs [9, 10]. The ongoing collaboration with the Gaza Cancer Registry (GCR) was conducted by the National Cancer Institute “Centro di Riferimento Oncologico”, Aviano (notheastern Italy); the Italian network of cancer registries (AIRTUM); and the Italian National Health Institute (ISS), Rome.

Herein, we describe the general characteristics and the crude survival experience of patients diagnosed with BC or with CRC between 2005 and 2014 in the Gaza Strip. Furthermore, as selected clinical data were available only for cases diagnosed in 2005–2006, we estimated the risks of death for patients with BC or CRC according to type of therapy, disease, grade, or stage.

## Methods

### Study population

We described the general characteristics and the cancer survival experience of people living in the Gaza Strip, diagnosed with BC or CRC in 2005–2014, according to the information recorded in the population-based GCR. Cancer Registries are identified as collectors of personal data for surveillance purposes without the need of explicit individual consent. The approval of a research ethic committee is not required because neither direct nor indirect intervention on patients took place. Nonetheless, the General Director of the Primary Health Care, MoH, (Dr. Fouad Elissawi) cleared the use of the registry data for study purposes. The data collection process used by GCR is an active one, carried on by GCR trained personnel who regularly visit the pathology departments and oncology clinics to collect newly detected cases and to update the already recorded ones. The update of the vital status is manually checked by means of the death registry.

For the aims of this analysis, to ensure data validity, each case was reviewed by a member of the GCR and co-author of this article (FE). The vital status and – eventually – the date of death were ascertained from the death registration database at Palestinian MoH. The last follow-up time was December 31st, 2016. Overall, from 2005 to 2014, 1495 women were diagnosed with BC (no cases of BC were recorded in men during the study period), and 878 people were diagnosed with CRC. This analysis was restricted to 1360 BC and 722 CRC patients after exclusion of: cases lacking the full date of birth (7 BCs and 21 CRCs); children under 15 years of age (2 BCs and 3 CRCs); and those patients with coincident dates of diagnosis and death (126 cases of BC and 132 cases of CRC).

Information on therapy, grade, and stage of disease was available for cases diagnosed in 2005–2006 only (i.e., 178 cases of BC and 80 cases of CRC). Accordingly, for these cases a multivariate analysis was conducted to estimate the risk of death.

### Statistical methods

The crude survival time was calculated as the time elapsed from date of cancer diagnosis to date of death, or to end of follow-up –whichever came first. At univariate analysis, the survival time for the totality of BC or CRC patients diagnosed from 2005 to 2014 was estimated by means of the Kaplan-Meier method [11].

For cases diagnosed in 2005–2006 only, a multivariate analysis was carried out to statistically assess the role of selected clinical variables on survival. To this end, hazard ratios (HRs) for all-cause mortality, and the corresponding 95% confidence intervals (95% CIs), were estimated using the Cox proportional hazard model adjusted for age at diagnosis (< 35, 35–44, 45–54, 55–64, 65–74, 75+ years) and gender, as appropriate [12]. The proportional hazard assumption was assessed through Schoenfeld residuals, including interactions with follow-up time [12].

## Results

### Breast cancer

**Fig. 1:**
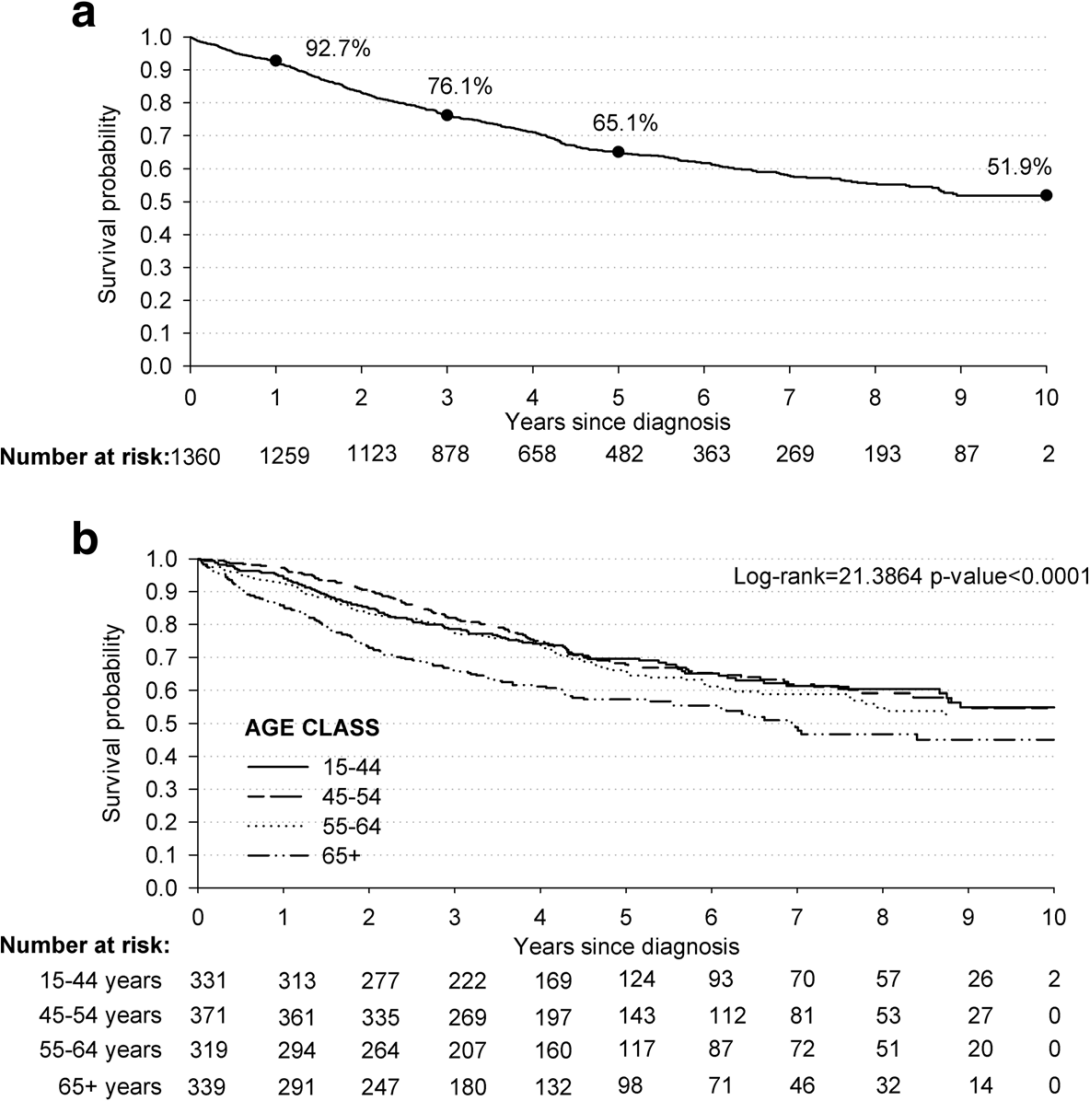
Kaplan-Meier estimates of survival probabilities among cases 1360 women diagnosed with breast cancer: overall (**a**) and according to age class (**b**). Gaza Strip, 2005–2014

The median age of the 1360 women diagnosed with BC in the Gaza Strip between 2005 and 2014 was 55.1 years (Inter Quartile Range -IQR: 45.8–64.8 years). The absolute number of cases more than doubled, from 178 in 2005–2006 up to 396 in 2013–2014 (Table 1), with slight variations in median ages –from 53.0 years (in 2005–2006) to 55.4 years (in 2013–2014). Overall, 76.1% (95% CI: 73.7–78.3) of these women was alive after 3 years, 65.1% (95% CI: 62.1–67.4) after 5 years, and 51.9% (95% CI: 47.9–55.7) after 10 years from BC diagnosis (Fig. 1a). The probability of survival after BC was strongly influenced by age, with women aged 65 years or older showing the lowest survival rates (i.e., 66.0% after 3, 57.4% after 5, and 45.1% after 10 years from diagnosis) (*p* < 0.001) (Fig. 1b).

**Table 1:** Description of breast and colorectal cancer incident cases diagnosed from 2005 to 2014 in the Gaza Strip

	Breast cancer		Colorectal cancer	
	Cases *N* = 1360	Deaths *N* = 486 (%)	Cases *N* = 722	Deaths *N* = 361 (%)
Sex				
Female	1360	486 (35.7)	343	169 (49.3)
Male	–	–	379	192 (50.7)
Age at cancer diagnosis (years)				
≤ 44	331	108 (32.6)	97	33 (32.0)
45–54	371	120 (32.4)	163	69 (40.5)
55–64	319	112 (35.1)	221	114 (49.3)
≥ 65	339	146 (43.1)	241	145 (59.3)
Calendar year at cancer diagnosis				
2005–2006	178	93 (52.3)	80	58 (72.5)
2007–2008	207	73 (35.3)	124	53 (47.7)
2009–2010	223	101 (45.3)	136	72 (52.9)
2011–2012	356	143 (40.2)	165	91 (55.2)
2013–2014	396	76 (19.2)	217	87 (40.1)

Selected clinical data available for the 178 women with BC diagnosed in 2005–2006 are discussed in detail (Table 2). The majority of them (60.1%) was diagnosed with an advanced stage of disease, but no difference was noted between the percentage of women diagnosed with well or moderately differentiated BC and those diagnosed with a poorly differentiated or undifferentiated disease. Among these 178 patients with BC, 83.7% underwent two or more therapies, in particular surgery (87.6%) and chemotherapy (76,4%).

**Table 2:** Hazard ratios (HR) of all-cause deaths, with corresponding 95% confidence intervals (CI), among 178 incident breast cancer cases diagnosed in 2005–2006 in the Gaza Strip according to clinical characteristics

	Cases	Deaths (*N* = 93)	
	N	N (%)	HR (95% CI)^a^
Age at diagnosis (years)			
< 55	98	47 (48.0)	1^b^
≥ 55	80	46 (57.5)	1.39 (0.93–2.09)
Stage ^c^			
Localized	33	10 (30.3)	1^b^
Regional/Distant	107	57 (53.3)	1.93 (0.98–3.80)
Missing	38	26 (68.4)	2.32 (1.10–4.91)
Grade			
Well and Moderately differentiated	75	31 (41.3)	1^b^
Poorly differentiated and Undifferentiated	70	42 (60.0)	1.67 (1.04–2.69)
Missing	33	20 (60.6)	1.33 (0.72–2.44)
Surgery ^c^			
No	18	14 (77.8)	1^b^
Yes	156	76 (48.7)	0.29 (0.16–0.53)
Chemotherapy ^c^			
No	38	23 (60.5)	1^b^
Yes	136	67 (49.3)	0.58 (0.36–0.94)
Radiotherapy ^c^			
No	81	46 (56.8)	1^b^
Yes	93	44 (47.3)	0.89 (0·57–1·39)
Hormone therapy ^c^			
No	109	63 (57.8)	1^b^
Yes	65	27 (41.5)	0.53 (0.33–0.84)
Number of therapies ^c^			
0–1	25	20 (80.0)	1^b^
2	55	29 (52.7)	0.32 (0.18–0.58)
3	56	23 (41.1)	0.25 (0.13–0.48)
4	38	18 (47.4)	0.26 (0.13–0.51)

^a^ Estimated using the Cox proportional hazard model adjusted for age; ^b^ Reference category; ^c^ The sum does not add up to the total because of missing values

**Fig. 2:**
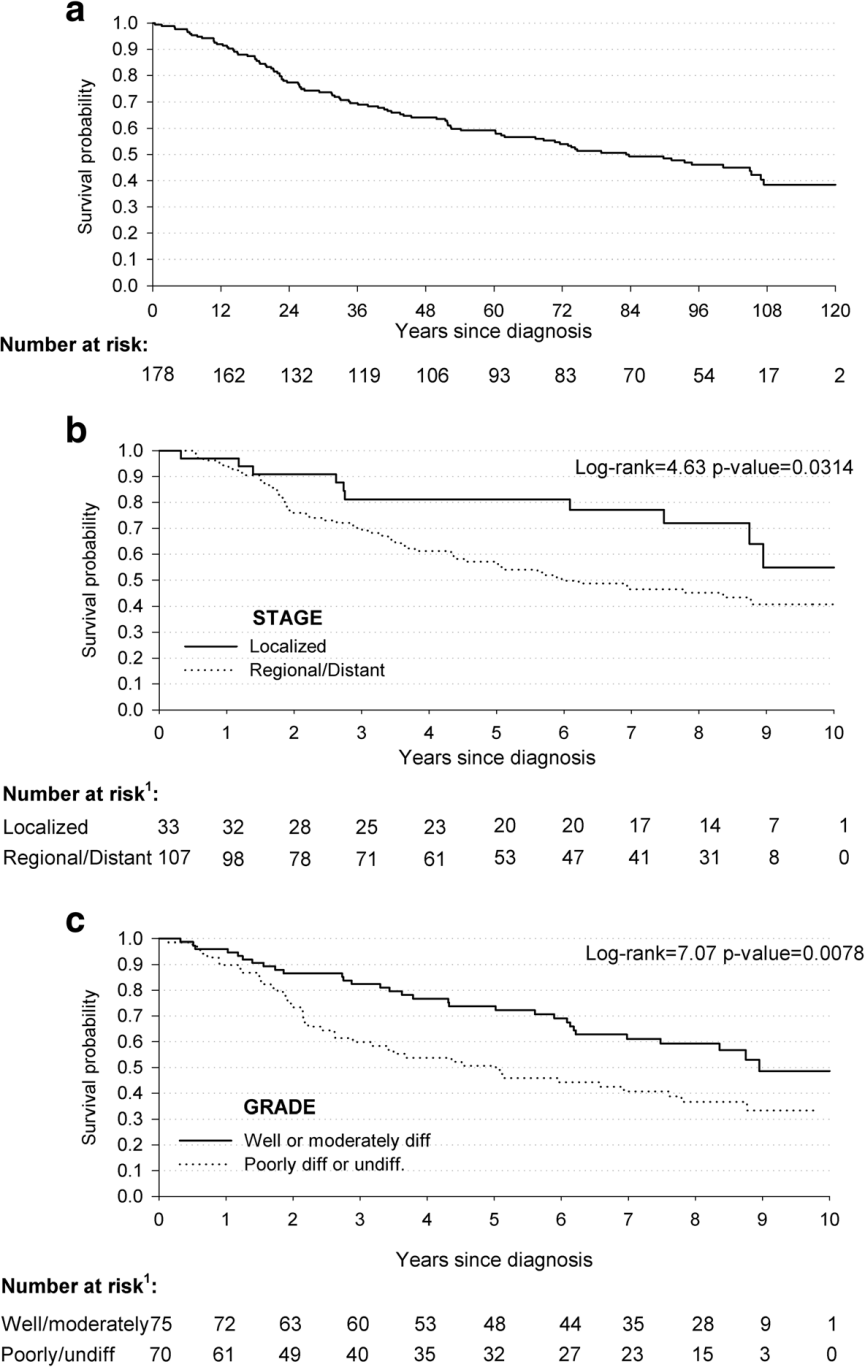
Kaplan-Meier estimates of survival probabilities among 178 women diagnosed with breast cancer: overall (**a**); according to stage (**b**); and grade (**c**). The Gaza Strip, 2005–2006. ^1^The sum does not add up to the total because of missing values

The 178 women diagnosed with BC between 2005 and 2006 were followed-up to December 31st, 2016 for a median period of 63.3 months (IQR: 23.0–100.3 months). During such period, 93 of them (52.2%) died, and 85 were censored. The estimated median survival time was 83.7 months (95% CI: 61.3–106.9) (Fig. 2a). Grade and stage of disease influenced the prognosis. Indeed, the survival probabilities of women with advanced stage of disease were statistically lower than those with a localized BC stage (p of log-rank test = 0.0314) (Fig. 2b). Concerning HR, advanced stage of disease was associated with an elevated risk of death as compared with those with a localized disease – of borderline statistical significance – (HR = 1.93, 95% CI: 0.98–3.80). Likewise, the survival probabilities stratified by grade of disease were different from each other (*p*-value = 0.0078) (Fig. 2c), and women diagnosed with poorly or undifferentiated BC were at 1.67-fold higher risk of death than women with well/moderate grade of cancer (HR:1.67, 95% CI:1.04–2.69) (Table 2).

Surgically treated women (87.6%) had the best prognosis, and those treated with two or more anti-cancer therapies presented a statistically significant 70% reduction in the risk of death, as compared with those who had little (i.e., only one type of treatment) or no access (2.9%) to anti-cancer therapies (Table 2).

### Colorectal cancer

**Fig. 3:**
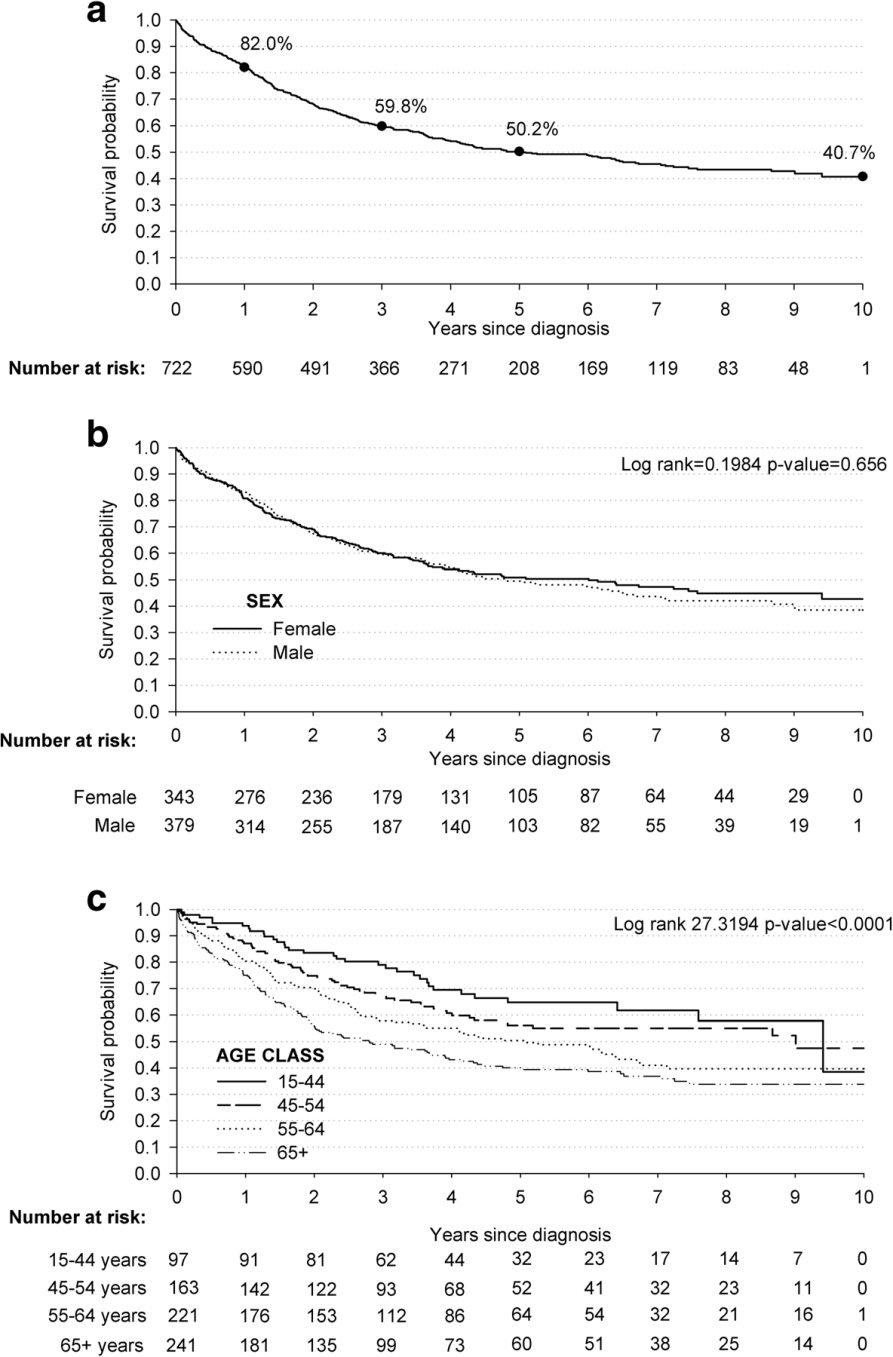
Kaplan-Meier estimates of survival probabilities among cases 722 cases diagnosed with colorectal cancer: overall (**a**), according to sex (**b**) and age class (**c**). Gaza Strip, 2005–2014

The median age of the 722 individuals (47·5% women) diagnosed with CRC in the Gaza Strip between 2005 and 2014 was 59.5 years (IQR: 51.2–68.6 years), and the absolute number of cases ranged from 80 in 2005–2006 to 217 in 2013–2014 (Table 1). The median ages at CRC diagnoses remained stable over time (58.7 years in 2005–2006, 59.9 years in 2013–2014). Overall, 59.8% (95% CI: 56.0–63.3) of them were  alive after 3 years, 50.2% (95% CI: 46.3–54.0) after 5 years, and 40.7% (95% CI: 35.6–45.8) after 10 years from diagnosis (Fig. 3a). The survival probabilities after a CRC diagnosis were not influenced by sex (Fig. 3b). Conversely, survival after CRC diagnosis was strongly influenced by age, with patients aged 65 years or older showing the lowest survival rates (i.e., 49.0% after 3, 40.1% after 5, and 33.8% after 10 years from diagnosis) (*p* < 0.001) (Fig. 3c).

Women accounted for 60.0% of the 80 patients diagnosed in 2005–2006 with a CRC (Table 3). Advanced stage of disease was documented in 49 out of 80 patients (61.3%), and a well or moderately differentiated grade of cancer was documented in 53 out of 80 patients (66.3%). As per clinical protocols, 88.8% of these 80 cases diagnosed in 2005–2006 underwent surgery, 87.5% received chemotherapy, and only 27.5% radiotherapy. More than three quarters of patients underwent two or more therapeutic regimens (Table 3).

**Table 3:** Hazard ratios (HR) of all-cause deaths, with corresponding 95% confidence intervals (CI), among 80 cases of colorectal cancers diagnosed in 2005–2006 in the Gaza Strip according to clinical characteristics

	Cases	Deaths (*N* = 46)	
	N	N (%)	HR (95% CI)^a^
Age at diagnosis (years)			
< 60	44	23 (52.3)	1^b^
≥ 60	36	23 (63.9)	1.38 (0.77–2.48)
Sex			
Male	32	20 (62.5)	1
Female	48	26 (54.2)	0.88 (0.48–1.59)
Stage ^c^			
Localized	27	10 (37.0)	1^b^
Regional/Distant	49	35 (71.4)	3.38 (1.57–7.29)
Grade ^c^			
Well and moderately differentiated	53	23 (43.4)	1^b^
Poorly differentiated and Undifferentiated	22	19 (86.4)	3.57 (1.87–6.81)
Surgery			
No	9	9 (100.0)	1^b^
Yes	71	37 (52.1)	0.27 (0.13–0.62)
Chemotherapy			
No	10	8 (80.0)	1^b^
Yes	70	38 (54.3)	0.37 (0.16–0.87)
Radiotherapy			
No	58	38 (65.5)	1^b^
Yes	22	8 (36.4)	0.24 (0.10–0.56)
Number Therapies			
0–1	18	16 (88.9)	1^b^
2	40	22 (55.0)	0.36 (0.17–0.74)
3	22	8 (36.4)	0.11 (0.04–0.31)

^a^ Estimated using Cox proportional hazard model adjust for sex and age; ^b^ Reference category; ^c^ The sum does not add up to the total because of missing values

**Fig. 4:**
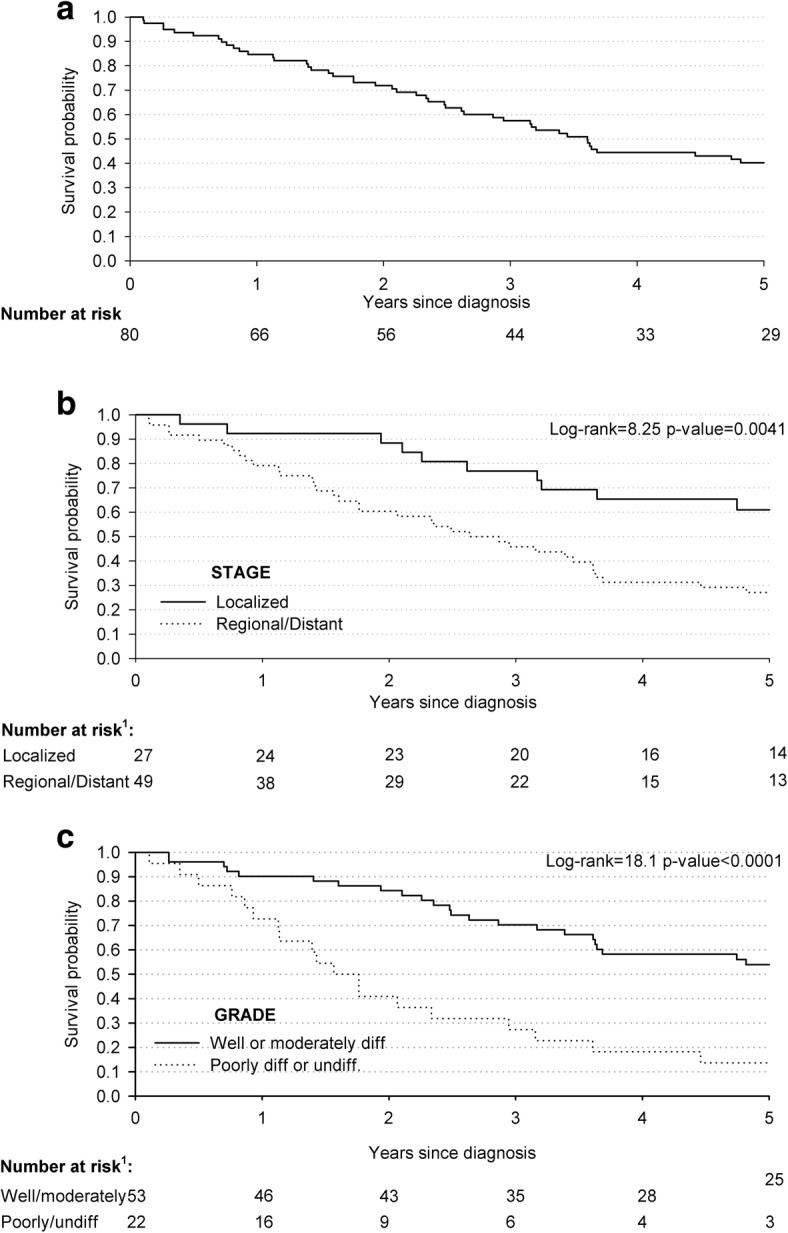
Kaplan-Meier estimates of survival probabilities among 80 cases diagnosed with colorectal cancer: overall (**a**); according to stage (**b**); and grade (**c**). The Gaza Strip, 2005–2006. ^1^The sum does not add up to the total because of missing values

Figure 4a shows the overall survival of patients with CRC diagnoses, the median survival time was 43.3 months (95% CI: 31.4–60.0), and 34 patients were censored. Cases with a localized disease had a higher survival rate (i.e., 61.0% 5-years survival) than those with regional/distant disease (i.e., 27.1% 5-years survival) (*p*-value = 0.0041) (Fig. 4b). Furthermore, CRC patients with a regional/distant stage of disease had an elevated risk of death, as compared to those with a localized disease (HR = 3.38, 95% CI: 1.57–7.29) (Table 3). Similarly, survival rates between patients with a well/moderate grade of CRC (i.e., 53.9% 5-years survival) and those with a poorly or undifferentiated grade (i.e., 13.6% 5-years survival) were significantly different (p-value< 0.0001) (Fig. 4c). Likewise, CRC cases diagnosed with poorly or undifferentiated CRC were at 3.57-fold (95% CI:1.87–6.81) higher risk of death than cases with well/moderate grade of disease (Table 3).

Patients who had been treated with two or more anti-cancer therapies presented a reduction in the risk of death (HR = 0.36 for those who received 2 out of three modalities; HR = 0.11 for cases who underwent all 3 types of treatments), as compared with those who had little (i.e., only one type of treatment) or no access (one patient) to anti-cancer therapies (Table 3).

## Discussion

Female BC and CRC are among the most common cancers diagnosed every year worldwide in both more- and less-developed WHO regions [13]. These cancers are very common also in the Gaza Strip, where the present study attempted -for the first time- to estimate the survival of people diagnosed with cancer in the Gaza Strip. Our findings indicate that 65.1% of women with BC, and 50.2% of patients with CRC were  alive after 5 years from diagnosis. In the subgroup of patients with available clinical information, the majority of patients were diagnosed at an advanced stage.

In agreement with our results, a Jordanian study disclosed a five-year survival for BC patients of 59.3%, showing that grade and stage had a significant effect on survival rates [14]. Mean age at breast cancer diagnosis was similar in Jordan [14], in Egypt [15], and in the Gaza Strip. The median survival time after BC in Egypt (i.e., 83.8 months) was equal to the estimate in the Gaza Strip (i.e., 83.7 months) [15] in 2005–2006. Furthermore, in Uganda 5-year survival probability was between 50 and 60% after a BC diagnosis, and in particular women with the luminal B sub-type had a 5-year survival around 30%. A possible explanation of these results could be the small sample size [16].

Concerning survival after CRC, a study conducted in Israel among Bedouin Arab and Jewish patients with CRC is worth mentioning [17]. The five-year overall survival was about 65% in both ethnic groups. However, the mean age at diagnosis was lower for the Bedouin Arab population (i.e., 57 years) than for the Jewish population (i.e., 69 years), pointing to a survival disadvantage for the Bedouin Arab ethnic group [17]. The 40% five-year survival for patients living in the Gaza Strip indicated a greater disadvantage in comparison with both Israeli ethnic groups.

The comparison of data from the GCR with those from highly-developed countries highlighted substantial differences. In the Gaza Strip, the  percentage of BC patients with localized disease at diagnosis was about half than that recorded in most European countries, and similar to the picture described in eastern European countries [18]. For CRC cases, the proportion of localized diseases in the Gaza Strip was about two-fold higher than that documented by Italian cancer registries [19]. With respect to treatment, the proportion of patients in the Gaza Strip who underwent chemotherapy and/or radiotherapy was higher than the proportion of Italian patients (88% vs. 39 and 28% vs. 10%, respectively).

In contrast with widely available estimates of cancer incidence rates, survival estimates at population level in less developed countries -including the WHO EMR- are less common [20]. The 5-year raw survival after BC varied substantially, from 38.8% in Setif (Algeria) [20] to 71.1% in Izmir, Turkey, in women diagnosed from 1995 to 1997 and followed-up to 2003, or to 61.3% in Saudi Arabia, among women diagnosed in 1994–1996 and followed-up to 2001 [21]. Similar variations emerged, at population level for survival after CRC diagnosis, from a raw 5-year survival of nearly 23% in Setif to 52% in Izmir [20].

Among the study strengths, the survival of cancer patients living in the Gaza Strip was assessed at a population level, while other studies were previously conducted in clinical setting [22, 23]; moreover, we described the heterogeneity survival. On the other hand, accuracy and completeness of data collection, in this study, might have suffered of potential limitations. With regards to the accuracy of information, the data from the GCR may have suffered of limitations due to the socio-economic situation and to the conflicts in the Gaza Strip, which may have limited the activity of the health personnel working in the West Bank and in Gaza. Furthermore, because of cultural taboos (e.g., negative and false perception toward cancer patients with a consequent isolation from family members), cancer patients tend to conceal their disease, which hinders their access to local hospitals. Concerning completeness, although cancer registration in the Gaza Strip started in 1996, it still faces several obstacles such as lack of appropriate hardware and software, insufficient staff, and training of health personnel. All these concerns represent key issues in the accurate assessment of the cancer burden in the Gaza Strip.

Notwithstanding this lack of completeness, our study results represent one of the first attempts to provide updated indications on the state of oncologic health care in the Gaza Strip. Moreover, it tries to address the problematic cancer care situation in this population.

The closure policy of the Gaza Strip for security reasons has caused an isolation of Gaza citizens. This isolation has affected the possibility to have adequate cancer care for many cancer patients living in the Gaza Strip. In particular, the denial or delay of permits to travel outside Gaza Strip for cancer patients referred to its two neighbouring countries (namely, Israel and Egypt) limits the opportunity of adequate diagnosis and/or treatment. Moreover, a number of antineoplastic medications are denied to patients due to the embargo [5, 24].

## Conclusions

In view of this already acknowledged difficult social, political, and economical context, the results from the present oncologic investigation further stresses the need to thoroughly re-assess and overcome the obstacles to a proper delivery of health care to the people living in the Gaza Strip. It is the scope of the continuing collaboration between Italian cancer registries and the GCR to contribute in supplying updated oncologic data from the Gaza Strip.

## Abbreviations

BC: Breast cancer
CIs: Confidence intervals
CRC: Colorectal cancer
EMR: Eastern Mediterranean region
GCR: Gaza Cancer Registry
HRs: Hazard ratios
IQR: Inter Quartile Range
MoH: Ministry of Health
OPT: Occupied Palestinian Territory
WHO: World Health Organization
